# Impact of COVID-19 visitation policies and hospital capacity on discharge readiness in medicine patients

**DOI:** 10.1007/s44250-023-00060-8

**Published:** 2023-11-30

**Authors:** Andrea S. Wallace, Sonja E. Raaum, Erin Phinney Johnson, Angela P. Presson, Chelsea M. Allen, Mackenzie Elliott, Alycia A. Bristol, Catherine E. Elmore

**Affiliations:** 1 The University of Utah College of Nursing, Annette Poulson Cumming Building Rm 5345, 10 South 2000 East, Salt Lake City, UT 84112-5880 USA; 2 University of Utah School of Medicine, Salt Lake City, USA

**Keywords:** Care transitions, Discharge planning, Health care quality, COVID-19

## Abstract

**Background:**

COVID-19 impacted the experience of being hospitalized with the widespread adoption of strict visitation policies to ensure healthcare worker safety. One result was decreased time of caregivers at the bedside of hospitalized patients.

**Objective:**

To understand the impact of pandemic-related system effects on patient-reported discharge preparation.

**Design:**

This mixed methods study included interviews with a sample of discharged patients during April 2020, and quantitative hospital data from April 2020 to February 2021.

**Participants:**

616 patients completed a measure of discharge readiness on their day of discharge and 38 patients completed interviews about their discharge experiences.

**Main measures:**

Readiness for discharge (RHDS), visitation policies, ward structure changes, COVID-19-unit census, time into the COVID-19 pandemic, patient characteristics (age, sex, race/ethnicity), admission type (planned/unplanned, for COVID-19), and discharge destination (home, home health, skilled nursing).

**Key results:**

Adult patients aged 30–45 (vs. young and older adult patients) and those being discharged to places other than home (e.g., skilled nursing facility) or to out-of-state residences report lower readiness (*p* < 0.05) on RHDS. Patient interviews revealed some gaps in discharge communication but, overall, patients expressed high discharge readiness and few concerns about how COVID-19 system changes impacted their discharge preparation.

**Conclusions:**

While there is some evidence that visitation policies and unit census may impact patient perceptions of discharge preparation, personal characteristics contributed more significantly to discharge readiness than system changes during COVID-19. Participant interviews demonstrated agreement, as most participants were discharged home and identified strong personal feelings of readiness for discharge.

*Clinical trials registration*: ClinicalTrials.gov ID NCT04248738, https://clinicaltrials.gov/ct2/show/NCT04248738.

**Supplementary Information:**

The online version contains supplementary material available at 10.1007/s44250-023-00060-8.

## Introduction

Beginning in March 2020, hospitals experienced immediate decreases in hospitalizations that then rapidly rebounded to pre-pandemic levels by summer 2020 [1]. While ED visits then decreased, hospital admissions increased due to sicker patients requiring admission [2]. As a result of these rapid shifts in hospitalizations, additional pressure was put on staffing and discharge throughput [3, 4]. By April 2020, nearly all academic hospitals had developed new respiratory isolation units and described disruption in typical rounding structures that included health professionals rounding separately: 90% of sites decreased in-room encounters across all provider types [5]. As a result, team disruptions may have led to decreased communication and collaboration around discharge planning [6].

Meanwhile, for patients and families, the experience of being hospitalized was directly impacted during COVID-19 by visitation policies [7]. Restrictive policies ensured healthcare worker (HCW) safety but presented a trade-off of decreased time of caregivers—unpaid family or friends—at the bedside of hospitalized patients. Importantly, high-quality discharge planning includes patients and their caregivers and is critical to understanding barriers to safe discharge and the effectiveness of discharge teaching [8–11]. Actively engaging caregivers in hospital discharge processes is associated with as much as a 25% decrease in hospital readmissions [12, 13].

While it is likely that COVID-19 pandemic conditions created system changes to the structure and processes of care—such as rapid shifts in unit census, medical management of a new and deadly infectious disease, and changes to patient visitation policies—that adversely affected the quality of discharge preparation and created additional barriers to patients’ ability to recover in the community after discharge, there is little information about whether or how the system changes related to the pandemic impacted the quality of discharge preparation, and which patients may have been most impacted. In this study, we sought to understand the impact of COVID-19 through qualitative interviews with patients who were discharged into a locked-down community, and quantitative analyses exploring how COVID-19 changes within the hospital, personal characteristics, and discharge destination impacted patient-reported discharge readiness.

## Methods

This convergent mixed methods study [14] was part of a larger parent study aiming to improve the quality of discharge planning from a quaternary care academic health sciences center University Hospital in the Intermountain West. Interviews were conducted with a sample of discharged patients during April 2020, and quantitative analyses were conducted on hospital data collected between April 22, 2020 and February 9, 2021.

### Ethical approval

This research was performed in accordance with the principles of the Declaration of Helsinki. Approval was granted by the Ethics Committee of University of Utah. Informed consent was obtained from all participants included in the study.

### Measures

For the qualitative portion, we developed an interview guide to understand participants’ ability to engage in self-management post-discharge, and to explore whether and how the discharge planning process identified potential needs post-discharge and contributed to successful transitions home after hospitalization. (See Supplementary Material.)

For the quantitative portion, the primary outcome measure was the eight-item Readiness for Hospital Discharge Scale (RHDS) [15–19] completed by the patient, assessing four dimensions of readiness that independently and mutually serve as outcomes of discharge preparation: perceptions of physical readiness to care for self (through recognition of availability of supportive persons and resources), knowledge (through education of a support person who can reinforce the information), ability to cope at home, and the level of expected support (having someone to help at home). The RHDS is correlated with quality of discharge teaching; difficulties coping post-discharge; care coordination and utilization of post-discharge services; lower perception of readiness at discharge is associated with increased use of both informal and formal support post-discharge; and hospital readmissions [18–23]. The RHDS uses a 0–10 point rating scale for each item; higher scores indicate greater readiness. A total mean score of less than 7 indicates low readiness and greater risk of readmission [16].

### Setting and sample

Interview participants were identified from those discharged from medical and surgical hospital units in March 2020, who were at least 18 years of age, spoke English (English need not be the first language), were able to communicate verbally, previously completed the RHDS, and indicated a willingness to be interviewed. Interviews occurred until data saturation was reached.

The quantitative sample were patients discharged by general internal medicine (GIM) teams during the first months of the COVID-19 pandemic (April 22, 2020–February 9, 2021). Patients were at least 18 years of age, spoke English (English need not be the first language), and were able to communicate verbally. Because of the implications for unique discharge processes, patients admitted due to a primary or secondary psychiatric diagnosis; enrolled in palliative/hospice care; residing in a skilled nursing facility (SNF) upon admission; overseen by transplant or bariatric surgical services; seen for unfunded end stage renal disease; and/or prisoners were excluded from this study.

#### Procedures

After discharge orders were placed into the electronic health record, clinical research coordinators approached patients in person (prior to March 14th) or via their hospital room phone to comply with COVID-19 safety precautions after March 14th. The IRB-approved informed consent script was completed with each patient. Consenting patients then verbally completed the RHDS. All qualifying patients on participating units were called, but many were not in their rooms or had already been discharged by the time the call was placed.

Patients who completed the RHDS in March 2020 and indicated they were willing to participate in a follow-up interview, were contacted via telephone in April 2020. Semi-structured interviews took approximately 30 min. Interview participants received a $10 gift card after completion of the interview. Interviews were audio-recorded and professionally transcribed.

Finally, unit occupancy data and dates related to patient visitation policy changes were retrospectively collected from hospital administrative records for all dates between April 22, 2020 and February 9, 2021.

#### Quantitative analysis

Numeric variables were summarized with mean and standard deviation (SD); categorical variables are summarized with number and percent. Univariable linear regression models of the RHDS and its 4 subdomains were done for all variables. Multivariable linear regressions were done with analyses exploring hypothesized interactions between discharge destination, admission for COVID-19, and visitor policy. All variables were included in the multivariable models except for variables that we decided to exclude a priori due to possible collinearity with other variables in the models (unit COVID proportion occupied, unit proportion of COVID patients, and admission type). In addition, as less than 7 indicates low readiness and greater risk of readmission, a dichotomized RHDS score was also created to allow for a sensitivity analysis using logistic regression for determining the factors associated with a high readiness for discharge.

Some levels of race and ethnicity were combined for regression analysis due to low counts. Long-term acute care (LTAC) was collapsed into the “other discharge destination” category in regression models, which also included acute rehabilitation facility, homeless shelter, COVID-19 hotel, and “left against medical advice”. Unit Census Percentage Occupied was based on total occupancy divided by capacity, while Unit COVID-19 Percentage Occupied was based on the number of COVID-19 patients on the unit divided by capacity. Thus, a 40-bed unit with 20 patients, 4 of whom are COVID-19 patients, would be at 50% Census Percentage Occupied and 10% COVID-19 Percentage Occupied. For these variables, “Proportion” instead of “Percentage” indicates that the decimal value is used instead of the percentage value (e.g. 0.5 rather than 50). Day of Pandemic was based off how many days had passed since the hospital enacted pandemic measures on March 14, 2020. Data collection for this study was briefly halted at that time and restarted on April 22, 2020, so the “Day of Pandemic” variable for this dataset begins on day 39. For models, this variable was converted to “Month of Pandemic” to aid in interpretation of the coefficients. Estimates, 95% confidence intervals (CI), and *p*-values are reported; statistical significance is defined as *p*-values less than 0.05. All analyses were conducted using R v4.0.3.

#### Qualitative analysis

Transcripts were reviewed for accuracy before coding. Authors A.B., S.R., K.F., and K.S. engaged in coding of the transcripts. Deductive codes were initially created based upon the interview guides [24]. Additional inductive codes emerged during coding, were discussed with the team, and then added to the codebook. After coding was completed, we used a thematic analysis process of reviewing codes to discuss and identify key themes [25]. Disagreements were documented and discussed during weekly meetings until consensus was reached. Trustworthiness criteria [24] were addressed to support rigor throughout data collection and analysis. Credibility was addressed by peer review and weekly team meetings. Additionally, team members reviewed each other’s coding and engaged in reflexivity by discussing biases and assumptions, to ensure accuracy across coding. An audit trail was maintained throughout data collection and analysis.

## Results

The quantitative sample included 638 unique GIM patients who completed the RHDS on the day of discharge. Twenty-two readmission events were excluded, bringing the total sample analyzed to 616 first-visit observations. See Table 1 for full demographic information about this sample and the additional 38 patients who completed interviews.

**Table 1:** Summary statistics

System characteristic	Type/level	Summary
Unit census percentage occupied	[Median (IQR)]	67.5 (57.5, 80.6)
Unit COVID percentage occupied	[Median (IQR)]	0 (0, 15)
Unit percentage of COVID patients (of total unit)	[Median (IQR)]	0 (0, 40)
Day of pandemic	[Median (IQR)]	224 (137.8, 282)
Total patient sample N = 616	Type/level	Summary
Patient age	18–30	81 (13%)
	31–45	180 (29%)
	46–60	194 (31%)
	61–75	148 (24%)
	76–90	13 (2%)
Patient sex	Female	316 (51%)
	Male	300 (49%)
Patient race	Non-White	94 (15%)
	Black or African-American	17 (2%)
	Asian	13 (2%)
	Native Hawaiian or Other Pacific Islander	19 (3%)
	American Indian	45 (7%)
	Two or more races	15 (2%)
	White	489 (79%)
	Unknown	35 (6%)
Patient ethnicity	Non-Hispanic	505 (82%)
	Hispanic	77 (12%)
	Unknown	34 (6%)
COVID-19 admission	Non-COVID-19 admission	433 (70%)
	COVID-19 admission	182 (30%)
Visitor policy	1 visitor	379 (62%)
	No VISITORS	237 (38%)
Discharge destination	Home	418 (68%)
	Home health	124 (20%)
	LTAC	3 (< 1%)
	Other	13 (2%)
	SNF	58 (9%)
Admission type	Elective (planned)	28 (5%)
	Urgent/emergent (unplanned)	588 (95%)
Patient readiness for hospital discharge—total	[Mean (SD)]	8.4 (1.5)
	[Range]	(1.6, 10.0)
RHDS high- and low-risk readmission groups	High (RHDS ≥ 7)	519 (84%)
	Low (RHDS < 7)	97 (16%)
Personal status (RHDS subdomain)	[Mean (SD)]	8.0 (1.8)
	[Range]	(0, 10.0)
Knowledge (RHDS subdomain)	[Mean (SD)]	8.2 (2.4)
	[Range]	(0, 10.0)
Perceived coping ability (RHDS subdomain)	[Mean (SD)]	8.7 (1.6)
	[Range]	(0, 10.0)
Expected support (RHDS subdomain)	[Mean (SD)]	8.7 (2.5)
	[Range]	(0, 10.0)
Patient interview sample N = 38	Type/level	Summary
Patient age	18–30	1 (2.6%)
	31–45	7 (18.4%)
	46–60	10 (26.3%)
	61–75	16 (68.4%)
	76–90	1 (2.6%)
Patient sex	Female	23 (60.5%)
	Male	13 (34.2%)
	Unknown	2 (5.2%)
Patient race	Asian	1 (2.6%)
	American Indian	1 (2.6%)
	White	34 (89.5%)
	Unknown	2 (5.2%)
Patient ethnicity	Non-Hispanic	36 (94.7%)
	Unknown	2 (5.2%)
Discharge destination	Home	33 (86.8%)
	Home health	4 (10.5%)
	SNF	1 (2.6%)

Missing values: Unit Census Percentage Occupied = 50, Unit COVID Percentage Occupied = 50, COVID = 1, PCDCS—Post-Discharge Coping Difficulties Scale = 507

### Quantitative results

RHDS total and subscale scores were most affected by patients’ discharge destination; among non-COVID admissions, those discharged to home health (− 0.48 (− 0.95, − 0.02), *p* = 0.041) or SNF (− 1.39 (− 2.04, − 0.74), *p* < 0.001) versus the patient’s own home reported lower overall readiness scores (Table 2). This finding was confirmed in our sensitivity analysis in which RHDS was dichotomized as high vs low readiness (Table 3). However, on subscale scores, among non-COVID admissions, those discharged to SNF had lower personal status scores (− 1.79 (− 2.58, − 1.00), *p* < 0.001, Table 4) and lower knowledge scores (− 1.80 (− 2.88, − 0.71), *p* < 0.001, Table 5), but higher perceived coping ability (− 1.62 (− 2.34, − 0.91), *p* < 0.001, Table 6) when compared to those discharged to their own homes.

**Table 2:** Contributors to patient readiness for hospital discharge scale (RHDS) total score variance

Variable	Univariable estimate (95% CI)	*p*-value	Multivariable estimate (95% CI)	*p*-value
Unit COVID proportion occupied	0.36 (− 0.33, 1.06)	0.31		
Unit proportion of COVID patients (of total unit)	0.17 (− 0.15, 0.48)	0.31		
Admission type				
Elective (planned)	–Reference–			
Urgent/emergent (unplanned)	− 0.46 (− 1.03, 0.11)	0.11		
Unit census proportion occupied	0.64 (− 0.19, 1.47)	0.13	0.95 (− 0.07, 1.96)	0.067
Month of pandemic	0.03 (− 0.01, 0.08)	0.13	0.02 (− 0.07, 0.10)	0.71
Age				
18–30	–Reference–		–Reference–	
31–45	− 0.40 (− 0.79, − 0.00)	0.048	− 0.46 (− 0.87, − 0.06)	0.025
46–60	− 0.34 (− 0.73, 0.05)	0.085	− 0.22 (− 0.63, 0.19)	0.29
61–75	− 0.34 (− 0.74, 0.07)	0.10	− 0.10 (− 0.53, 0.33)	0.65
76–90	− 0.02 (− 0.90, 0.86)	0.97	0.06 (− 0.82, 0.93)	0.90
Sex				
Female	–Reference–		–Reference–	
Male	− 0.17 (− 0.40, 0.07)	0.17	− 0.10 (− 0.35, 0.14)	0.41
Race/ethnicity				
White non-Hispanic	–Reference–		–Reference–	
Hispanic	0.13 (− 0.23, 0.50)	0.47	0.01 (− 0.38, 0.40)	0.95
Non-White/two or more races	− 0.14 (− 0.44, 0.16)	0.36	− 0.13 (− 0.46, 0.20)	0.44
COVID				
Non-COVID admission	–Reference–		–Reference–	
COVID admission	0.20 (− 0.06, 0.46)	0.13	0.12 (− 0.27, 0.52)	0.53
Visitor policy				
1 visitor	–Reference–		–Reference–	
No visitors	− 0.12 (− 0.36, 0.12)	0.33	0.14 (− 0.39, 0.67)	0.61
Discharge destination				
Home	–Reference–		–Reference–	
Home health	− 0.12 (− 0.41, 0.17)	0.42	− 0.48 (− 0.95, − 0.02)	0.041
Other	− 1.02 (− 1.74, − 0.30)	0.006	− 0.82 (− 1.70, 0.06)	0.067
SNF	− 1.29 (− 1.68, − 0.89)	< 0.001	− 1.39 (− 2.04, − 0.74)	< 0.001
COVID:discharge destination				
Non-COVID admission:home			–Reference–	
COVID admission:home health			0.75 (− 0.02, 1.52)	0.058
COVID admission:other			− 2.29 (− 4.20, − 0.38)	0.019
COVID admission:SNF			0.94 (− 0.20, 2.07)	0.11
Visitor policy:discharge destination				
1 visitor:home			–Reference–	
No visitors:home health			0.25 (− 0.42, 0.93)	0.46
No visitors:other			1.26 (− 0.64, 3.16)	0.19
No visitors:SNF			− 0.31 (− 1.17, 0.55)	0.48

SNF = Skilled Nursing Facility. Missing Values: Unit Census Percentage Occupied = 50, COVID = 1; One or more predictors missing: 51 observations

**Table 3:** Contributors to RHDS dichotomized categories of high (≥ 7) and low (< 7) discharge readiness

Variable	Univariable OR (95% CI)	*p*-value	Multivariable OR (95% CI)	*p*-value
Unit COVID proportion occupied	1.39 (0.39, 4.98)	0.62		
Unit proportion of COVID patients (of total unit)	1.22 (0.67, 2.23)	0.51		
Admission type				
Elective (planned)	–Reference–			
Urgent/emergent (unplanned)	0.00 (0.00, Inf)	0.98		
Unit census proportion occupied	2.32 (0.53, 10.15)	0.26	0.84 (− 1.08, 2.75)	0.39
Month of pandemic	1.04 (0.96, 1.12)	0.34	0.00 (− 0.16, 0.17)	0.98
Age				
18–30	–Reference–		–Reference–	
31–45	0.47 (0.21, 1.07)	0.072	− 0.89 (− 1.78, − 0.00)	0.049
46–60	0.55 (0.24, 1.26)	0.16	− 0.41 (− 1.33, 0.50)	0.37
61–75	0.66 (0.28, 1.57)	0.35	− 0.08 (− 1.07, 0.91)	0.87
76–90	0.60 (0.11, 3.22)	0.55	− 0.45 (− 2.22, 1.31)	0.61
Sex				
Female	–Reference–		–Reference–	
Male	0.68 (0.44, 1.06)	0.087	− 0.28 (− 0.76, 0.20)	0.25
Race/ethnicity				
White Non-Hispanic	–Reference–		–Reference–	
Hispanic	1.01 (0.50, 2.03)	0.97	− 0.15 (− 0.95, 0.64)	0.70
Non-White/two or more races	0.63 (0.38, 1.05)	0.076	− 0.56 (− 1.13, 0.01)	0.054
COVID				
Non-COVID admission	–Reference–		–Reference–	
COVID admission	1.18 (0.72, 1.91)	0.51	0.15 (− 0.51, 0.82)	0.65
Visitor policy				
1 visitor	–Reference–		–Reference–	
No visitors	0.87 (0.56, 1.36)	0.54	− 0.08 (− 1.02, 0.87)	0.87
Discharge destination				
Home	–Reference–		–Reference–	
Home Health	0.95 (0.53, 1.71)	0.87	− 0.28 (− 0.90, 0.34)	0.37
Other	0.33 (0.11, 1.00)	0.049	− 1.08 (− 2.22, 0.06)	0.063
SNF	0.29 (0.16, 0.53)	< 0.001	− 1.51 (− 2.20, − 0.82)	< 0.001

The odds ratio (OR) is the ratio of the odds of being of moderate to high readiness to the odds of being of low readiness. SNF = Skilled Nursing Facility. Missing Values: Unit Census Percentage Occupied = 50, COVID-19 = 1; One or more predictors missing: 51 observations

**Table 4:** Contributors to personal status (RHDS subdomain) score variance

Variable	Univariable estimate (95% CI)	*p*-value	Multivariable estimate (95% CI)	*p*-value
Unit COVID proportion occupied	0.68 (− 0.15, 1.50)	0.11		
Unit proportion of COVID patients (of total unit)	0.32 (− 0.06, 0.70)	0.10		
Admission type				
Elective (planned)	–Reference–			
Urgent/emergent (unplanned)	− 0.46 (− 1.15, 0.22)	0.18		
Unit census proportion occupied	0.11 (− 0.88, 1.10)	0.83	0.47 (− 0.75, 1.69)	0.45
Month of pandemic	0.03 (− 0.02, 0.08)	0.24	0.02 (− 0.09, 0.12)	0.74
Age				
18–30	–Reference–		–Reference–	
31–45	− 0.50 (− 0.97, − 0.03)	0.037	− 0.57 (− 1.06, − 0.08)	0.022
46–60	− 0.41 (− 0.87, 0.06)	0.086	− 0.27 (− 0.76, 0.23)	0.29
61–75	− 0.28 (− 0.76, 0.21)	0.27	− 0.07 (− 0.60, 0.45)	0.78
76–90	0.31 (− 0.74, 1.36)	0.56	0.61 (− 0.44, 1.67)	0.25
Sex				
Female	–Reference–		–Reference–	
Male	0.12 (− 0.16, 0.41)	0.40	0.18 (− 0.12, 0.48)	0.24
Race/ethnicity				
White Non-hispanic	–Reference–		–Reference–	
Hispanic	0.28 (− 0.15, 0.72)	0.20	0.21 (− 0.27, 0.68)	0.39
Non-White/two or more races	0.27 (− 0.09, 0.63)	0.15	0.32 (− 0.07, 0.72)	0.11
COVID				
Non-COVID admission	–Reference–		–Reference–	
COVID admission	0.34 (0.03, 0.65)	0.034	0.06 (− 0.42, 0.53)	0.81
Visitor policy				
1 visitor	–Reference–		–Reference–	
No visitors	− 0.16 (− 0.45, 0.13)	0.27	0.05 (− 0.59, 0.69)	0.88
Discharge destination				
Home	–Reference–		–Reference–	
Home health	− 0.26 (− 0.62, 0.09)	0.15	− 0.51 (− 1.07, 0.05)	0.074
Other	− 1.21 (− 2.09, − 0.32)	0.008	− 1.49 (− 2.56, − 0.43)	0.006
SNF	− 0.98 (− 1.47, − 0.49)	< 0.001	− 1.79 (− 2.58, − 1.00)	< 0.001
COVID:discharge destination				
Non-COVID admission:home			–Reference–	
COVID admission:home health			0.70 (− 0.23, 1.64)	0.14
COVID admission:other			− 1.64 (− 3.95, 0.67)	0.16
COVID admission:SNF			2.24 (0.87, 3.62)	0.001
Visitor policy:discharge destination				
1 visitor:home			–Reference–	
No visitors:home health			− 0.01 (− 0.82, 0.80)	0.98
No visitors:other			2.55 (0.25, 4.84)	0.030
No visitors:SNF			0.69 (− 0.34, 1.73)	0.19

SNF = Skilled Nursing Facility. Missing Values: Unit Census Percentage Occupied = 50, COVID = 1; One or more predictors missing: 51 observations

**Table 5:** Contributors to knowledge (RHDS subdomain) score variance

Variable	Univariable estimate (95% CI)	*p*-value	Multivariable estimate (95% CI)	*p*-value
Unit COVID proportion occupied	− 0.02 (− 1.17, 1.12)	0.97		
Unit proportion of COVID patients (of total unit)	0.08 (− 0.43, 0.60)	0.75		
Admission type				
Elective (planned)	–Reference–			
Urgent/emergent (Unplanned)	− 0.06 (− 0.99, 0.86)	0.89		
Unit census proportion occupied	0.83 (− 0.54, 2.20)	0.23	1.08 (− 0.60, 2.77)	0.21
Month of pandemic	0.03 (− 0.04, 0.10)	0.38	− 0.01 (− 0.16, 0.13)	0.85
Age				
18–30	–Reference–		–Reference–	
31–45	− 0.11 (− 0.75, 0.53)	0.75	− 0.16 (− 0.83, 0.52)	0.65
46–60	− 0.15 (− 0.79, 0.48)	0.64	− 0.01 (− 0.69, 0.67)	0.98
61–75	− 0.69 (− 1.35, − 0.03)	0.042	− 0.40 (− 1.12, 0.32)	0.27
76–90	− 0.87 (− 2.30, 0.56)	0.23	− 0.85 (− 2.31, 0.61)	0.25
Sex				
Female	–Reference–		–Reference–	
Male	− 0.21 (− 0.59, 0.18)	0.29	− 0.20 (− 0.61, 0.21)	0.34
Race/ethnicity				
White Non-Hispanic	–Reference–		–Reference–	
Hispanic	− 0.07 (− 0.66, 0.53)	0.83	− 0.34 (− 1.00, 0.31)	0.31
Non-White/two or more races	− 0.35 (− 0.85, 0.14)	0.16	− 0.50 (− 1.04, 0.05)	0.074
COVID				
Non-COVID admission	–Reference–		–Reference–	
COVID admission	0.14 (− 0.28, 0.57)	0.51	0.12 (− 0.53, 0.78)	0.71
Visitor policy				
1 visitor	–Reference–		–Reference–	
No visitors	− 0.14 (− 0.54, 0.26)	0.49	− 0.11 (− 0.99, 0.78)	0.81
Discharge destination				
Home	–Reference–		–Reference–	
Home Health	− 0.24 (− 0.72, 0.23)	0.32	− 0.62 (− 1.40, 0.15)	0.12
Other	− 1.00 (− 2.18, 0.18)	0.098	− 0.95 (− 2.41, 0.52)	0.21
SNF	− 2.20 (− 2.85, − 1.55)	< 0.001	− 1.80 (− 2.88, − 0.71)	0.001
COVID:discharge destination				
Non-COVID admission:home			–Reference–	
COVID admission:home health			0.84 (− 0.45, 2.12)	0.20
COVID Admission:other			− 2.16 (− 5.34, 1.02)	0.18
COVID Admission:SNF			− 0.01 (− 1.90, 1.88)	> 0.99
Visitor policy:discharge destination				
1 visitor:home			–Reference–	
No visitors:home health			0.57 (− 0.55, 1.69)	0.32
No visitors:other			2.13 (− 1.04, 5.30)	0.19
No visitors:SNF			− 0.70 (− 2.13, 0.73)	0.34

SNF = Skilled Nursing Facility. Missing Values: Unit Census Percentage Occupied = 50, COVID-19 = 1; One or more predictors missing: 51 observations

**Table 6:** Contributors to perceived coping ability (RHDS subdomain) score variance

Variable	Univariable estimate (95% CI)	*p*-value	Multivariable estimate (95% CI)	*p*-value
Unit COVID proportion occupied	0.79 (0.03, 1.55)	0.043		
Unit proportion of COVID patients (of total unit)	0.31 (− 0.04, 0.65)	0.085		
Admission type				
Elective (planned)	–Reference–			
Urgent/emergent (unplanned)	− 0.34 (− 0.96, 0.29)	0.29		
Unit census proportion occupied	0.27 (− 0.64, 1.18)	0.56	0.48 (− 0.63, 1.59)	0.40
Month of pandemic	0.05 (0.00, 0.09)	0.046	0.01 (− 0.08, 0.11)	0.80
Age				
18–30	–Reference–		–Reference–	
31–45	− 0.40 (− 0.83, 0.04)	0.073	− 0.46 (− 0.91, − 0.02)	0.042
46–60	− 0.28 (− 0.71, 0.15)	0.20	− 0.16 (− 0.61, 0.29)	0.48
61–75	− 0.27 (− 0.72, 0.17)	0.23	0.08 (− 0.40, 0.55)	0.75
76–90	− 0.14 (− 1.11, 0.82)	0.77	0.06 (− 0.90, 1.02)	0.90
Sex				
Female	–Reference–		–Reference–	
Male	− 0.07 (− 0.33, 0.20)	0.62	− 0.02 (− 0.29, 0.25)	0.88
Race/ethnicity				
White Non-Hispanic	–Reference–		–Reference–	
Hispanic	0.17 (− 0.23, 0.57)	0.40	0.05 (− 0.38, 0.48)	0.82
Non-White/two or more races	0.08 (− 0.25, 0.42)	0.62	0.18 (− 0.18, 0.54)	0.34
COVID				
Non-COVID admission	–Reference–		–Reference–	
COVID admission	0.32 (0.04, 0.60)	0.028	0.10 (− 0.34, 0.53)	0.66
Visitor policy				
1 visitor	–Reference–		–Reference–	
No visitors	− 0.25 (− 0.52, 0.02)	0.067	− 0.07 (− 0.65, 0.51)	0.81
Discharge destination				
Home	–Reference–		–Reference–	
Home health	− 0.26 (− 0.58, 0.06)	0.11	− 0.61 (− 1.13, − 0.10)	0.019
Other	− 1.21 (− 2.01, − 0.41)	0.003	− 1.54 (− 2.51, − 0.58)	0.002
SNF	− 1.38 (− 1.81, − 0.94)	< 0.001	− 1.62 (− 2.34, − 0.91)	< 0.001
COVID:discharge destination				
Non-COVID admission:home			–Reference–	
COVID admission:home health			0.59 (− 0.25, 1.44)	0.17
COVID admission:other			− 0.55 (− 2.64, 1.55)	0.61
COVID admission:SNF			1.38 (0.14, 2.63)	0.030
Visitor policy:discharge destination				
1 visitor:home			–Reference–	
No visitors:home health			0.25 (− 0.49, 0.99)	0.51
No visitors:other			1.94 (− 0.15, 4.03)	0.068
No visitors:SNF			− 0.33 (− 1.27, 0.61)	0.49

SNF = Skilled Nursing Facility. Missing Values: Unit Census Percentage Occupied = 50, COVID-19 = 1; One or more predictors missing: 51 observations

From the interaction between COVID-19 diagnosis and discharge destination we find that relative to non-COVID-19 admissions, COVID-19 patients discharged to a SNF rated their personal status [2.24 (0.87, 3.62), *p* < 0.001, Table 4] and coping ability [1.38 (0.14, 2.63), *p* = 0.030, Table 6] higher at discharge compared to those discharged to their own home. In contrast, relative to non-COVID-19 admissions those with a COVID-19 diagnosis and discharged to “other destination” reported significantly lower ratings for expected support [− 4.81 (− 7.98, − 1.63), *p* = 0.003, Table 7] compared to those discharged to their own home. From the interaction between discharge destination and hospital visitor policy, personal status varied by visitor policy where, relative to when one visitor was allowed, during a no visitation policy, those discharged to “other destination” reported higher personal status [2.55 (0.25, 4.84), *p* = 0.030, Table 4] when compared to those discharged to their own home.

**Table 7:** Contributors to expected support (PT-RHDS subdomain) score variance

Variable	Univariable estimate (95% CI)	*p*-value	Multivariable estimate (95% CI)	*p*-value
Unit COVID proportion occupied	0.01 (− 1.12, 1.14)	0.98		
Unit proportion of COVID patients (of total unit)	− 0.04 (− 0.56, 0.48)	0.87		
Admission type				
Elective (planned)	–Reference–			
Urgent/emergent (unplanned)	− 0.98 (− 1.91, − 0.05)	0.039		
Unit census proportion occupied	1.33 (− 0.02, 2.68)	0.053	1.75 (0.07, 3.44)	0.042
Month of pandemic	0.02 (− 0.05, 0.09)	0.56	0.05 (− 0.10, 0.19)	0.50
Age				
18–30	–Reference–		–Reference–	
31–45	− 0.59 (− 1.23, 0.05)	0.073	− 0.67 (− 1.34, 0.01)	0.053
46–60	− 0.53 (− 1.16, 0.11)	0.10	− 0.45 (− 1.13, 0.23)	0.20
61–75	− 0.12 (− 0.78, 0.54)	0.72	0.00 (− 0.71, 0.72)	0.99
76–90	0.63 (− 0.80, 2.06)	0.39	0.40 (− 1.06, 1.86)	0.59
Sex				
Female	–Reference–		–Reference–	
Male	− 0.52 (− 0.91, − 0.13)	0.008	− 0.37 (− 0.78, 0.04)	0.073
Race/Ethnicity				
White Non-Hispanic	–Reference–		–Reference–	
Hispanic	0.15 (− 0.45, 0.74)	0.63	0.13 (− 0.52, 0.79)	0.69
Non-White/two or more races	− 0.56 (− 1.05, − 0.07)	0.027	− 0.52 (− 1.06, 0.03)	0.062
COVID				
Non-COVID admission	–Reference–		–Reference–	
COVID admission	0.00 (− 0.42, 0.43)	0.99	0.22 (− 0.43, 0.88)	0.50
Visitor policy				
1 visitor	–Reference–		–Reference–	
No visitors	0.07 (− 0.33, 0.47)	0.72	0.68 (− 0.20, 1.57)	0.13
Discharge destination				
Home	–Reference–		–Reference–	
Home health	0.28 (− 0.21, 0.77)	0.27	− 0.19 (− 0.96, 0.59)	0.63
Other	− 0.66 (− 1.88, 0.56)	0.29	0.70 (− 0.77, 2.16)	0.35
SNF	− 0.59 (− 1.26, 0.09)	0.087	− 0.35 (− 1.44, 0.73)	0.52
COVID:discharge destination				
Non-COVID admission:home			–Reference–	
COVID admission:home health			0.86 (− 0.43, 2.14)	0.19
COVID admission:other			− 4.81 (− 7.98, − 1.63)	0.003
COVID admission:SNF			0.12 (− 1.77, 2.01)	0.90
Visitor policy:discharge destination				
1 visitor:home			–Reference–	
No visitors:home health			0.21 (− 0.92, 1.33)	0.72
No visitors:other			− 1.57 (− 4.74, 1.59)	0.33
No Visitors:SNF			− 0.90 (− 2.33, 0.53)	0.22

SNF = Skilled Nursing Facility. Missing Values: Unit Census Percentage Occupied = 50, COVID-19 = 1 One or more predictors missing: 51 observations

Age additionally influenced readiness scores, where adults 31–45 reported the lowest total readiness levels [− 0.46 (− 0.87, − 0.06), *p* = 0.025, Table 2] compared to the young adults 18–30, a finding that was also confirmed in our sensitivity analysis using the dichotomized RHDS total score (Table 3). Perceived coping ability after discharge varied by age where, again, those age 31–45 reported lower levels than the youngest adults (18–30) [− 0.46 (− 0.91, − 0.02), *p* = 0.042, Table 6]. No additional differences were found between age categories.

Finally, the only evidence of a system-level factor contributing to variability in discharge readiness ratings was for the expected support subscale, where increased percent occupancy was associated with increased expected support scale scores [1.75 (0.07, 3.44), *p* = 0.042, Table 7].

### Qualitative results

Across interviews, participants identified three key themes related to their experiences of hospital-to-home transitions during the pandemic: (1) perceptions of discharge process; (2) influence of personal resources at home; (3) post-discharge impact of COVID-19. (See Table 8 for themes and exemplar quotes).

**Table 8:** Qualitative themes and exemplar quotes

Theme	Exemplar quotes
Theme 1: Perceptions of Discharge Process During COVID	“They were good about getting me the information, if they didn't have it right at hand. They made sure that they got answers that I was curious about.” (#003)
	“They just gave me the discharge sheet saying I have phone numbers to call and what to look for in case there's complications from surgery, that kind of thing.” (005)
	“They didn’t really tell me what to expect, really. And you know, it’s still been going on, and I still don’t really know what to expect.” (015)
	“They didn’t tell me what 3 months from now is gonna look like.” (038)
Theme 2: Influence of Personal Characteristics	“I think that I'm self motivated enough that I do my own therapy. I really didn't need somebody coming in and making sure I did it. Because it's important to me to have pretty much full mobility.” (#004)
	“None of this stuff applies to me. I mean, I had my husband here and my daughter here. I don't need anyone else, and before that I was staying with family in Utah, so I don't need community services. I mean, it's different if I was elderly. I mean, I am elderly, but 80 and need it, didn't have anyone around, so I'm a different situation. I don't need any community services.” (#008)
	“Well, when they questioned me, I told them that I’d be okay, you know. Once I got home, I’d be taken care of and uh, I am the kinda person that just don’t give up because, you know, my health problems. I have to gain my strength back, so that’s what I did.” (#30)
	“It's hard to get a hold of my personal doctor out there and then just they stopped taking new appointments. Anything that I need, I'm in a different state. I first have to go through the military with everything and it's seems hard to get ahold of anyone in Utah…But I think my main issue has been the lack of follow-up from Utah and then not having any clear discharge instructions.” (010)
	“Unfortunately…my physical therapy I’ve only been able to get two sessions in here in my home. I live in, uh, four hours away from Salt Lake in Idaho and, um, our little community which is Blaine County has had the highest per capita COVID-19 cases in the nation, um, including New York City, um, just by per capita basis.” (014)
Theme 3: Post-discharge Impact of COVID	“Because you're not able to do it in person [therapy appointment] and they're being sent videos and just the accountability…The accountability. If you're feeling not into it today, you're like, ‘Oh, three sets of 10. I'm going to do two sets of five. Who's going to know?’ You don't have somebody there pushing you and being like, ‘Nope’." (006)
	“And I was also supposed to follow up with my urologist when I got back home to make sure I didn't need stitches removed …and my local urologists and urogynecologists both are not even seeing patients in person. They only do on the phone interview and they couldn't help me if my stitches didn't dissolve. So I was sweating and having like a really bad anxiety for the … they didn't dissolve until six and a half weeks and it was close because I was told do not go, absolutely, do not go past 8 weeks, that they should have dissolved in 6 weeks or less. So that was really stressful.” (009)
	“On the positive side, my work hasn’t wanted me to come back. So, my leave has been extended. And that’s made it easier for me to follow through with therapy at home because I have nine more hours a day to be at home.” (022)
	“I would say no. It’s not a—it really hasn’t affected much” (#026)
	“I was feeling a little more old-school of actually having a doctor look at it, touch it, feel it, move it, bend it, to make sure it was looking good. But, you know, the Zoom meeting with the PA [physician assistant] um, seemed to uh, satisfy, you know, the doctor I was talking to—the PA I was talking to and so, they said everything looked okay. And I ended up having to take three stitches out myself.” (#031)
	“I didn’t plan on all my kids being home. I planned on having a little bit of downtime to just relax. So, I felt like maybe a little bit more. There’s been a few times, you know, I’ve overdone things because I’ve had to—just like in the last couple weeks where I’m like, oh, I need to do laundry, or I need to do this. You know, I’m up on my crutches doing things. Um, which I maybe would have had more time to do or just not having my kids home to do it.” (#036)
	“But I think it would have been—would I be doing—I think I’ve done really well. But would I be doing better had he [physical therapist] had [sp] the ability to come in the house two or three times a week or if I were able to go, you know, to um, rehab at the hospital. I’ve gotta think I would do somewhat better if I had, you know, that instruction and that discipline of somebody else. So, since, you know, COVID, it’s not possible, but it would have to slightly impact.” (#037)

#### Theme 1: Discharge process during COVID-19

During interviews, participants mostly shared positive experiences of receiving discharge education and information packets. However, several participants identified negative aspects of the discharge process such as instructions delivered while they were not completely aware or were on pain medication. Due to COVID-19 restrictions, one participant shared not receiving verbal instructions, only the printed packet of information. Multiple participants shared feeling ill-prepared to know what to expect after leaving the hospital, particularly after a few months had passed. Overall, while most participants identified receiving helpful discharge education, several shared examples of lacking verbal explanations and experienced limited time spent on discharge instructions.

#### Theme 2: Influence of personal resources at home

The social resources of interview participants influenced how participants described the discharge process. Most participants identified having support networks at home and shared positive feedback of the discharge process and ability to follow discharge instructions. A few participants shared initial difficulty in performing activities of daily living (such as toileting, showering, etc.) and decreased mobility which became easier after time. Moreover, when participants were asked if they had access to recommended community resources, most denied accessing supportive services or failed to identify a need for community resources since they had support at home.

The experiences above were in contrast to participants with fewer resources or from out-of-state, who shared increased difficulty post-discharge. For example, one participant received preparation for the long car ride but did not receive instructions regarding home care. Another shared that where they are living was experiencing higher rates of COVID-19 that limited ability to follow through with physical therapy appointments.

#### Theme 3: Post-discharge impact of COVID-19

The impact of COVID-19 was most clearly seen post-discharge. Several participants shared that COVID-19 restriction resulted in more in-home aid post-discharge or had positive effects on their work situation as they could recover and work from home. In contrast, others discharged to a home in a different state shared struggles accessing resources such as finding available outpatient follow-up and therapy appointments.

## Discussion

In this study, we explored the impact of COVID-19 system changes on patient-reported discharge readiness and transitions home during the pandemic. Our quantitative findings revealed that discharge destination most significantly impacts discharge readiness, rather than system changes during the pandemic, but that impact varies across different readiness constructs. Adult patients (vs. young and older adult patients) and those being discharged to places other than home or to out-of-state residences report lower readiness. For those discharged to a community setting other than to home or SNF, variability in the Personal Status discharge readiness subscale was additionally affected by the hospital visitor policy. In support of this finding, our interviews with participants who were primarily discharged to home revealed high personal readiness for discharge and coping. However, this group still noted gaps in discharge communication, homegoing preparation, and experienced barriers to needed follow-up care.

Contrary to what was expected, we found little evidence that the system changes occurring during Covid-19 impacted the overall readiness of patients discharged to home settings, even for those with an unplanned admission. While prior work suggested that higher unit census and larger size of units are associated with lower patient discharge readiness [21], the impact of unit on discharge readiness may be driven by additional intrinsic factors such as nursing staff level of education or experience that were not captured in our study. Conversely, it is possible that COVID-19 system changes had limited impact on those who were able to return home and had stable social network and resources, findings that align with other qualitative research conducted with patients from this parent study [26]. Further work to associate this with risk of readmission in the setting of COVID-19 and other system disruptions would help determine whether interventions can be tailored to prevent revisits/readmissions by avoiding inappropriate discharge location [27].

Across all scales, patients age 31–45 reported feeling less ready for hospital discharge. This suggests there are nuances to patient perception of readiness based on age, and aligns with prior work that found that those younger than 55 years were less ready for discharge on all subscales compared to those aged 55–64 [28]. It has been posited that age-based differences reflect the way that younger patients (< 50) are treated during the discharge process, where assumptions are made regarding the ability to cope at home resulting in less discharge teaching [8]. Our findings may also reflect the stress that this age range may be expecting in terms of balancing multiple competing priorities, such as work, family, and caregiving responsibilities upon returning home after hospitalization, particularly during the COVID-19 pandemic.

While the majority of prior data on patient readiness for discharge has been limited to patients returning home, one study reported that out of a population of readmitted patients, 28% felt unready to discharge on index hospitalization; this did not vary based on discharge destination [29]. Our data contradict these findings, suggesting that the discharge destination has an impact on the patient's readiness to be discharged and, thus, identifying a potential area for improving the quality of discharge support. Decisions to go to an institutional setting such as SNF or LTAC are driven by clinical and functional status, but can reflect a lack of support at home [30]. In the realities of clinical practice, discharge teaching regarding self-care is limited for patients discharged to home [21, 29] and may be much more limited for patients being discharged to institutional settings, with the assumption they will be receiving ongoing assistance and teaching as needed. However, the fact that non-COVID-19 patients discharged to SNF or LTAC report lower readiness across all readiness subscales likely shows a gap in support in discharge preparation. Similarly, interview participants who were discharged home, but lived in a different state than where they received hospital care, reported experiencing more post-discharge difficulties, which were compounded by COVID-19. Additional preparation may be needed for these patients, particularly when contact with their social networks is disrupted.

This study is limited in several ways. First, the important demographic variable of race/ethnicity was collapsed due to limited numbers represented across categories, and we did not have access to variables that may better indicate homegoing support such as marital status or clinical details such as length of stay or homegoing with medical devices that, in addition to demographic and social factors, have been shown to be associated with patient discharge readiness [31]. Further, while we believe that the system variables created capture contextual system disruptions during each discharge event, we were unable to include other potential variables indicating system disruptions and stressors during the pandemic. For example, given that discharge preparation is a core nursing function, and unit-based nurse staffing has been shown to impact RHDS, future studies should consider more comprehensively exploring whether and how nurse staffing strains during COVID-19 has impacted patient readiness. Additionally, interviews were conducted early in the pandemic with patients who were mainly discharged to home, limiting our ability to qualitatively explore the effect of discharge destination. Finally, we are unable to explain an outlying finding wherein increasing COVID-19 unit census contributed to improved expected support, suggesting the need to perhaps explore this variable and relationship in greater detail.

## Conclusion

We found little evidence that hospital census and visitor policies during COVID-19 impacted patient-reported discharge readiness for the larger population of patients discharged from medicine units. Rather, our analyses uncovered patient populations likely in need of additional support during hospital discharge when contact restrictions or staffing limit in-person anticipatory planning: adult (vs. younger adult and older adult patients) being discharged to institutional settings. As health systems and clinicians continue to learn from the experiences of COVID-19, efforts aimed at improving hospital discharge, care transitions, and post-discharge outcomes may wish to give focused attention on these populations reporting lower discharge preparation.

### Supplementary Information

Below is the link to the electronic supplementary material.

Supplementary file 1 (DOCX 16 kb)

## Data Availability

The deidentified data that support the findings of this study are available on request from the corresponding author. The data are not publicly available due to privacy or ethical restrictions.

## Declarations

### Relevant reporting checklist

Per EQUATOR Reporting Guideline Decision Tree, we used the COREQ checklist for reporting the Qualitative aspect of this mixed methods study.
