# Supplementary material for: Impact of COVID-19 visitation policies and hospital capacity on discharge readiness in medicine patients
Source: Discov Health Syst. 2023 Nov 30;2(1):45. doi: 10.1007/s44250-023-00060-8 (PMC10689550; doi:10.1007/s44250-023-00060-8)
Supplement: Supplementary file 1 — (DOCX 16 kb) [file 44250_2023_60_MOESM1_ESM.docx]

**Supplementary Material.**

NEEDS Study Patient Interview Guide

*Assessing Patients’ Outpatient Process of Self-Management related to their 1) condition, 2) physical and social environment, and 3) individual and family, keeping in mind the outcomes of self-management behaviors, health status, and quality of life.*

1. What kinds of help have you needed for self-care following discharge from the hospital?
   1. Have you had a family member of other caregiver in the home assisting you?
2. What community-based services (such as, groups that can help you with transportation, home care, medication information, or support) were you referred to in order to help you at home?
   1. Have you made use of those services?
3. When you were in the hospital, how well did you feel like your doctors, nurses and physical therapists understood your needs at home?
   1. Who on the care team (e.g., nurse, case manager, surgeon) seemed to know what you needed best?
4. How well do you feel your discharge instructions matched your needs? Did the doctors and nurses seem to take your needs into account?
5. How well do you feel everything was communicated to you by your doctors and nurses?
   1. Do you feel like the treatment team was all on the same page or was there inconsistency in information that was conveyed to you?
6. Since you’ve returned home, how well have you been able to follow-through on your discharge instructions?
   1. Do you think your ability to recover well at home has been impacted by the COVID-19 crisis?

5 – Extremely, 4 – Very, 3 – Moderately, 2 – Slightly, 1 - Not at all

- - - How?
  1. Do you think your ability to recover well at home would have been impacted if you were discharged today?

5 – Extremely, 4 – Very, 3 – Moderately, 2 – Slightly, 1 - Not at all

- - - How?

1. We understand there are challenges coping with the Corona Virus (COVID-19) pandemic. Because of COVID-19, do you currently have needs in any of the following areas?
   1. Transportation to see a doctor
   2. Paying for medical care
   3. Paying for medicine
   4. Paying for food
   5. Someone to pick up medicine or food
   6. Paying for utility bills
   7. Paying for a place to live
   8. Someone to help you if you become ill
   9. Help with lost wages or help looking for work
   10. Help with child care or elder care
   11. I don’t have any of these needs at this time

Remind participants of community resources available to them.
